# ASPP2 involvement in p53-mediated HIV-1 envelope glycoprotein gp120 neurotoxicity in mice cerebrocortical neurons

**DOI:** 10.1038/srep33378

**Published:** 2016-09-14

**Authors:** Zhiying Liu, Yunjin Zang, Luxin Qiao, Kai Liu, Yabo Ouyang, Yulin Zhang, Dexi Chen

**Affiliations:** 1 Beijing You’an Hospital, Capital Medical University, Beijing, 100069, China; 2 Beijing Institute of Hepatology, Capital Medical University, Beijing, 100069, China; 3 The Affiliated Hospital of Qingdao University, Organ Transplantation Center, Qingdao City, Shandong Province, 266003, China

## Abstract

The mechanisms behind HIV-1-associated neurocognitive disorders are still unclear. Apoptosis-stimulating protein 2 of p53 (ASPP2) is a damage-inducible p53-binding protein that stimulates p53-mediated apoptosis and transactivates proapoptotic and cell cycle regulatory genes. It has been reported that ASPP2 has a specific regulatory function in the death of retinal ganglion cells and the development of Alzheimer’s disease. In this study, we used p53 and ASPP2 knockout mice and primary cerebrocortical neuron culture to analyze the role of the interaction between ASPP2 with p53 in HIV-1 envelope glycoprotein gp120-induced neurotoxicity. The results showed that 10 ng/mL gp120 protein might stimulate p53 overexpression and translocation to the nucleus, and 30 ng/mL gp120 protein could stimulate both p53 and ASPP2 translocation to the nucleus, but only with p53 overexpression. The primary cultured neurons of p53^−/−^ASPP2^+/−^ mice had a higher survival rate than p53^−/−^ mice under gp120 protein stress. The interaction of ASPP2 with p53 induced by a high dose of gp120 stimulated Bax transcription and contributed to caspase-3 cleavage, and ASPP2-siRNA attenuated gp120 induced neuron death through inhibition of Bax expression. These results suggest that ASPP2 plays an important role in p53-mediated neuronal apoptosis under gp120 stress.

In the combined antiretroviral therapy (cART) era, HIV-1 associated neurocognitive disorders (HAND) have become an independent risk factor for AIDS mortality^1^, and the total prevalence of HAND was approximately 37% in HIV-1 infected people^2^. A number of key mechanisms have been suggested for how HIV-1 leads to neurological dysfunction since the discovery of HAND^3^,^4^, but many issues have still not been clarified. Synaptic damage and neuronal apoptosis were found to be involved in the pathological changes in the brain tissue of HAND patients^5^. The mechanism of HIV-1-induced neuronal damage and apoptosis is very complicated. *In vitro* and *in vivo* studies have shown that HIV-1 envelope glycoprotein gp120, released from HIV-1 infected macrophages and glial cells, was confirmed to be able to cause nerve damage via target C-C chemokine receptor 5 (CCR5), C-X-C chemokine receptor 4 (CXCR4) or other cell surface receptors located at the neuron surface^6^,^7^,^8^,^9^. Our previous *in vitro* study also revealed that gp120 could induce neuronal neurite damage and neuronal apoptosis in mice^10^.

p53 plays a crucial role in stress-induced cell cycle arrest or apoptosis^11^, and it can be overexpressed in a variety of neurodegenerative diseases^12^,^13^. It was reported that p53 was upregulated in the glial cells of HAND patients^14^ and was a key protein in gp120-mediated neurotoxicity^15^. Although it is clear that the p53 regulates neuronal apoptosis, the relevant pathway of p53 overexpression and p53-mediated cell death signal transduction in neurons is still poorly understood. Apoptosis stimulation of p53 protein 2 (ASPP2) is a damage-inducible p53-binding protein that stimulates p53-mediated apoptosis and transactivates proapoptotic and cell cycle regulatory genes. ASPP2 interaction with p53 is an important mechanism for promoting apoptosis^16^,^17^. Experimental evidence from the ASPP2 regulation of p53 mainly comes from cancer research. Whether ASPP2 and p53 stimulate HIV-related neural degeneration still has not been reported. In this study, we investigated the role of ASPP2 and p53 on neurotoxicity in the presence of gp120 protein using primary neuronal culture of p53^−/−^ASPP2^+/−^ mice and p53^−/−^ mice *in vitro*.

## Materials and Methods

### Materials and animals

Wild-type BALB/C mice were purchased from the Chinese Military Academy of Medical Sciences. ASPP2 gene knockout mice and p53 gene knockout mice were gifted from Charles Lopez’s Lab, Oregon Health & Science University (OHSU). Animal experimental protocols were approved by the Institutional Animal Care and Use Committee at the Capital Medical University and conformed to the Capital Medical University guidelines for the care and use of animals in research. Mouse monoclonal antibodies against p53, p21 and Bax were purchased from Santa Cruz (Santa Cruz Biotechnology, CA, USA), Rabbit polyclonal antibody against active Caspase-3 was purchased from Abcam, Hong Kong, and rabbit polyclonal antibody against Alpha-tubulin was purchased from Sigma, Germany. Mouse monoclonal antibody against MAP-2, rabbit polyclonal antibody against GFAP, rabbit monoclonal antibody against ASPP2 and mouse monoclonal antibody against p53 for the Fluorescence Immunohistochemistry assay were all purchased from Sigma, Germany. Propidium iodine (PI) was purchased from Sigma, Germany. All primers were ordered from Life Technologies Corporation, USA. Eukaryotic expression gp120 protein was purchased from Sino Biological Inc,China. Mouse ASPP2 monoclonal antibody and p-CMV-ASPP2 plasmid were gifted from the Charles Lopez Lab at OHSU. The TUNEL detect kit was ordered from Roche Molecular, Switzerland. The DNA extraction kit was purchased from Qiagen China Co., Ltd. (Shanghai, China). Neuron culture reagents including Neurobasal, Penicillin–streptomycin, Glutamax and B27 were purchased from Gibco(Life Technologies Corporation, USA). Calcein AM was obtained from Molecular Probes (Life Technologies Corporation, USA). Conjugated Cy3 anti-mouse antibody was obtained from BioLegend, USA. The HRP-conjugated anti-mouse and anti-rabbit secondary antibodies were obtained from the Jackson Laboratory, USA. Total RNA extraction kit and RNeasy mini kit were obtained from Qiagen,Germany. SuperScript III First-Strand Synthesis System for RT-PCR was obtained from Invitrogen (Life Technologies Corporation, USA). The MTT Cell Proliferation Assay Kit was ordered from Thermo Fisher Scientific, USA.

### Primary neuron cultures

Primary cortical neuronal cultures were prepared from embryonic wild-type, homozygous or heterozygous knockout BALB/C mice at 16 days of gestation according to the previously described techniques^18^. In each group, 5 mice were used for the study of gp120 toxicity. Briefly, dissociated cells were plated onto precoated poly-Lysine plates. Cover glasses pretreated with acetone, absolute ethanol and 0.1 M HCl in turn were coated and used for immunofluorescence assays. Cells were maintained in the cultivation medium (Neurobasal medium supplemented with 50 U/50 μg/mL penicillin–streptomycin, 2.0 mM Glutamax and 2% B27) at 37 °C and 5% CO_2_ atmosphere. For the gp120 protein treatment study, a range of concentrations (from 5 ng/mL to 85 ng/mL) of recombinant gp120 protein was added to the culture medium on the eighth day at 37 °C for various times. The triplicate experiments were repeated to avoid experimental error, and the experiments were performed in a sterile hood with sterilized labware and solutions.

### Cell viability assay

Cell viability was assessed by PI and Calcein-AM uptake test or MTT cell proliferation assay^19^. In the PI and Calcein-AM uptake test, cultured neurons were incubated with 1 μg/mL Calcein–AM for 30 min; 10 μg/mL PI was then added for 15 min to the medium at 37 °C. Cells were counted using a fluorescent microscope emitting a wave length in the range of 485/645. Cell mortality is expressed as the ratio of PI-positive cells/calcein positive +PI positive cells. In the MTT assay, cell proliferation was detected using an MTT Cell Proliferation Assay Kit with the manufacturer’s protocol.

### TUNEL assay

Cultured neurons treated with gp120 on a cover slip were washed with phosphate-buffered saline (PBS) and then post fixed in 4% paraformaldehyde. Sections were washed three times in PBS and then permeabilized with 3% triton X-100 for 20 min followed by three washings in PBS. Two microliters TdT enzyme and 100 μl buffer from a TUNEL kit was added to the cover slip and incubated at 37 °C for 1 hour. The reaction was terminated by adding PBS washing buffer to the dish and washing three times for 45 min at room temperature. Cells were then counterstained with 4′,6-diamidino-2-phenylindole (DAPI) to assess nucleus morphology and were viewed on a fluorescence microscope.

### Isolation of protein

For primary culture neurons, each 35 mm dish had 90 μl nondenaturing RIPA buffer (10 mM Tris-HCl (pH 7.5), 1% sodium deoxycholate, 1% NP-40, 150 mM NaCl) and Protease/Phosphatase inhibitor mixture (0.25 mM PMSF, 1 ug/mL aprotinin, 1 μg/mL leupeptin, 1 μg/mL pepstatin, 50 mM NaF, 2 mM Na_3_VO_4_, 1 mM okadaic acid, 2 mM EGTA) added and incubated on ice for 30 minutes. After centrifuging at 23,000 *g* for 25 minutes at 4 °C, the supernatant was removed to a new tube and the protein concentration was detected using an ABC kit. Depending on its concentration, the protein was divided into 200 μg per tube and stored at −80 °C for detection of immunoblotting.

### Western blotting and immunoprecipitation

Briefly, 40 μg protein was resolved by 12% SDS-PAGE and then transferred to nitrocellulose membranes to detect ASPP2, p53, Bax, p21, Caspase-3 and tubulin proteins. The blots were blocked with TBS-T (20 mM Tris-HCl pH 7.6, 8.5% NaCl and 0.1% Tween-20) containing 5% nonfat dry milk at room temperature for 1 hour. The blots were then treated with related primary antibody in 1xTBS-T containing 3% bovine serum albumin at 4 °C overnight. After three washes with TBS-T buffer, the blots were incubated with secondary antibodies conjugated to HRP for 1 hour at room temperature. This process was followed by 4 washes with TBS-T. The final visualization of the target bands was produced using an enhanced chemiluminescence system (Pierce SuperSignal) on X-ray films following the manufacturer’s instruction. For immunoprecipitation, 500 μg protein was incubated with 2 μg of ASPP2 antibody for 3 h at 4 °C and then with protein A/G agarose for 2 h with gentle shaking. Beads were collected by centrifugation, washed 3 times with RIPA buffer, and eluted by incubation with SDS loading buffer at 95 °C for 5 min. The immunoblot assay was performed with anti-p53 monoclonal antibody.

### Fluorescence Immunohistochemistry

Primary cultured neurons on the cover slip were washed 3 times with PBS and then post fixed in 4% paraformaldehyde followed by ethanol/acetic acid (1:2, vol/vol) for 10 min. Sections were washed twice in PBS and then permeabilized with 3% Triton X-100 for 20 min followed by 3 washings in PBS. The sections were preblocked with 2% goat serum and 1% BSA in PBS for 2 hours at room temperature, followed by primary antibody (mouse against MAP-2, rabbit against GFAP, rabbit monoclonal antibody against ASPP2 and mouse monoclonal antibody against p53) at 4 °C overnight. The slides were washed 3 times with PBS and incubated with second antibodies (sheep Anti-Mouse IgG fragment–Cy3 antibody or chicken anti-rabbit IgG-FITC) for 1 hour at 37 °C. Then, the slides were washed as before, were then counterstained with DAPI to assess nucleus morphology and were viewed on a fluorescence microscope.

#### Assays of SSBR

DNA single-strand breaks were detected using DNA polymerase-I-mediated biotin-dATP nick translation labeling (PANT). Sections were postfixed in 4% polyformaldehyde for 10 min and permeabilized with 1% Triton X-100 for 10 min after washing. Sections were incubated in a moist air chamber at 37 °C for 90 min in a labeling mixture containing 10 M each of dGTP, dATP and dTTP, 10 M Cy3-dCTP, and 20 U/ml of *Escherichia coli* DNA polymerase-I (Sigma, St. Louis, MO) in reaction buffer for 30 min before the PANT. The reaction was terminated by washing the slides twice in PBS. To determine non-specific labeling, selected sections were incubated in the reaction buffer without the enzyme. The results of the PANT reflect the single strand break signal (SSB), and the stronger the signal, the weaker the SSBR capability.

### DNA fragment assay

Briefly, the cell culture media was thoroughly discarded, and then 200 μl bacterial resuspended buffer (mini plasmid prepare Kit from Promega) was added to the 35-mm dish. Resuspended cell solutions were moved from the dish to a 1.5 mL tube, and SDS was added to 0.5%, proteinase K to 2 μg/μl for cells lysis. The tube was incubated at 55 °C for 1 hour and then equal volumes of neutralization buffer were added. After centrifuging at 12,000 *g* for 15 min, the supernatant was transferred to a new tube, and an equal volume of Resin solution and 2.2 μl 20Xssc was added. DNA-resin solution was added to the column. After all solutions were passed through the column, a 4 mL washing buffer (75% Ac-OH, 0.1 x SSC) was used with the wash column. The DNA binding to resin was eluted by adding 40 μl of 70 °C H_2_O to the column and spun at 10,000 rpm for 1 min. The sample DNA solution was loaded to 1.2% TAE agarose gel and run at 80 V for 1.5 hour. The DNA fragment picture was obtained in UV light.

### Detection of mRNA levels with quantitative real-time PCR (qPCR)

An RNeasy mini kit was used to isolate total RNA according to the manufacturer’s instruction. Using the SuperScript III First-Strand Synthesis System for RT-PCR, 2.5 μg total RNA were reverse transcribed to cDNA in 50 μL RT reaction mix at 37 °C for 2 h, and 50 ng cDNA were used to perform qPCR. The nucleus housekeeping gene GAPDH was selected as the endogenous control. The primers for mouse GAPDH and Bax have been described in our previous study^10^. Briefly, the primers for the *bax* gene were 5′-GGGTGGTTGCCCTTTTCTACT-3′ (forward primer) and 5′-CCCGGAGGAAGTCCAGTGTC-3′ (reverse primer). For GAPDH, the sequences were 5′-CGTGGGGCTGCCCAGAACATC-3′ (forward primer) and 5′-GGATGACCTTGCCCACAGCCT-3′ (reverse primer). The primers for mouse p53, ASPP2 and p21 were designed as follows: 5′-GGT GTC ACG CTT CTC CGA AGA-3′ forward primer and 5′-ATCAGCAACTGGGCGTTCAGA-3′ reverse primer for p53, 5′-TTC CAT GCG GTT CGG GTC C-3′ forward primer and 5′-ATC AGC AAC TGG GCG TTC AGA-3′ reverse primer for ASPP2, and 5′-CGA AAT GCC CAG GAT AGT GTG-3′ and 5′-CGC CCA GAG TTA GTT ATA GGA-3′ for p21. All primers were synthesized by Invitrogen Life Technologies (Shanghai, China). p53, ASPP2, bax and p21 mRNA levels were estimated by qPCR using an SYBR Green PCR Kit (Takara, Dalian). The qPCR reactions were performed with a TaqMan 7900 HT Fast Real-time PCR System with our previously described primer concentrations and cycling parameters^10^. The relative mRNA expression of the target gene was normalized to the internal control gene GAPDH through comparing the sample’s ΔCT, and the fold change of the target mRNA was investigated by the 2^−ΔΔCT^ equation.

### Chip-PCR

To investigate the potential interaction of p53 and ASPP2 in the presence of gp120, CHIP-PCR was performed to detect the promoter binding region of p53 with p21 and bax, which were downstream genes of p53. Briefly, chromatin immunoprecipitation was performed as follows: cells were cross-linked with 500 μl l×PBS containing 1% formaldehyde solution and then quenched with glycine. After washing the cells with cold PBS 2 times, 500 μl cold PBS was added and centrifuged for 5 minutes at 7,000 rpm, the supernatant was discarded and 200 μl SDS lysis buffer containing 1% cocktail (Roche) was added and sonicated on ice by a sonifier ultrasonic cell disrupter (Shenggong bio-technology, Shanghai, China) with 30 × 30 s pulses followed by vortexing every five cycles. Two hundred microliters of RIPA ChIP buffer (10 mM Tris-HCl, pH 7.5, 140 mM NaCl, 1 mM EDTA, 0.5 mM EGTA, 1% Triton X-100, 0.1% SDS, 0.1% Na deoxycholate, 1% protease inhibitor and 1 mM PMSF) was added, and the extract was spun for 10 min at 10,000 rpm at 4 °C. The supernatant was collected into a new tube, and the pellet was re-extracted with another 200 μl of RIPA ChIP buffer. Then, immunoprecipitation was performed using protein G magnetic beads with 2 μg of either the anti-p53 antibody (Sigma, Germany) or the anti-ASPP2 antibody (Sigma, Germany) at 4 °C. The normal rabbit IgG (Cell Signaling technology, USA) was added as the input control. Finally, the ChIP buffer was incubated to reverse the crosslinks, then 750 μl PB buffer (Qiagen PCR purification kit, Germany) was added, and finally DNA was purified through a Qiaquick column and eluted into 50 μl H_2_O. The binding of the p53 protein with the p21 promoter region and bax promoter region were detected by PCR using the sample obtained from the CHIP directly. PCR reactions were performed by adding 2 μl of the eluted DNA, primers, Taq DNA Polymerase (Life Technology, USA) and dNTPs in a 25 μl volume following the procedure below: denaturation at 95 °C for 3 min followed by 40 cycles (95 °C for 30 s, 60 °C for 30 s and 72 °C for 15 s). The primer sequences specific for the p21 and bax promoter binding regions were as follows: 5′-CCA TCA CAG AAG AGG AGG C-3′ for the forward primer and 5′-CTG CTT TGG AGA AGC TGT GAG-3′ for the reverse primer for the p21 promoter binding site according to EL-Deiry’s reports^20^, 5′-CCGAGAGAGGACATCCGCGT-3′ for the forward primer and 5′-GCAAACAGACCCCCAG-3′ for the reverse primer for the bax promoter binding site of p53 according to Edward C Thornborrow’s report^21^.

### Statistical analysis

Statistical analysis was performed using SPSS or StatViewTM software with assistance from the biostatistics consulting services at the University of Washington. Dichotomized data were analyzed using a two-by-two contingency table and Fisher’s exact test. Student’s *t*-test was used when comparing one continuous variable for two groups. One-way analysis of variance (ANOVA) with post hoc comparison by Fisher’s LSD was performed for all experiments with >2 experimental groups.

## Results

### Gp120 induced neuron death in a dose- and time-dependent manner

**Figure 1:**
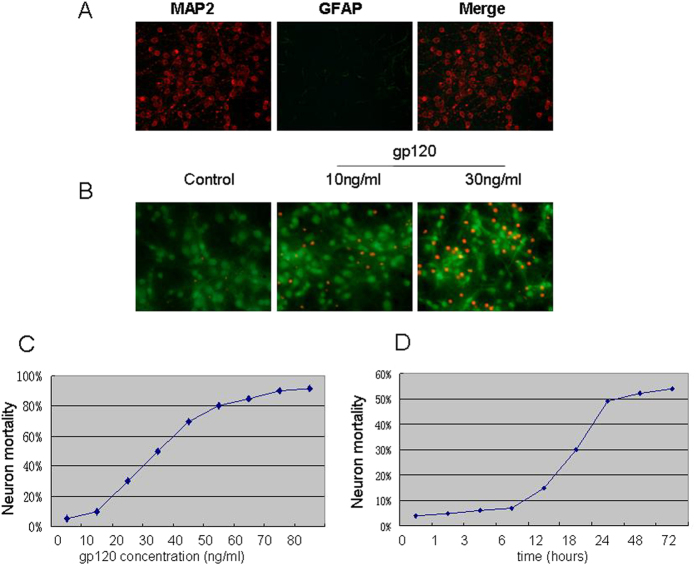
gp120 protein neurotoxicity. (**A**) The purity of primary cultured neurons was detected through immunofluorescence double staining using neuron-specific anti-MAP-2 antibody and glial cell-specific anti-GFAP antibody. (**B**) Morphologic change of neurons exposed to different gp120 protein levels with propidium iodine (PI) and Calcein-AM double staining. (**C**) The correlation of gp120 concentration and neuron apoptosis for 24 h. (**D**) The correlation of 30 ng/mL gp120 exposure time and neuronal apoptosis.

HIV-1 envelope protein gp120 can directly or indirectly induce neuronal apoptosis by interacting with chemokine receptors, CCR5 and CXCR4, expressed on the surface of neurons. In this study, we intended to identify the role of the interaction between ASPP2 and p53 in the mediation of gp120-induced neuronal injury in primary cultured neurons. Thus, it was necessary to assess the purity of the primary cultured neurons and the dose of gp120 that induced neuronal apoptosis. The purity of primary cultured neurons was detected through immunofluorescence double staining using neuron-specific anti-MAP-2 antibody and glial cell-specific anti-GFAP antibody. The results showed that the purity of cultured neurons was up to 95% (Fig. 1A). Neuronal cell mortalities under various doses of gp120 or exposure time were assessed by PI/Calcein-AM uptake test, and the results are shown in Fig. 1B–D. The results showed that the cell mortalities, as the ratio of PI-positive cells/(Calcein positive + PI positive cells), rose with the increase in gp120 concentration (treated for 24 h) and the prolonging of gp120 exposure time (with 30 ng/mL gp120 concentration). In detail, minor cell mortalities (less than 10%) were observed when treated with gp120 protein lower than 15 ng/mL, whereas a robust cell mortality were observed when treated with gp120 concentrations higher than 25 ng/mL. Among those, 40% cell mortality occurred when treated with 30 ng/mL for 24 h, and over 80% cell mortality occurred when treated with gp120 with concentrations over 55 ng/mL. In contrast, the rate of cell mortality was less than 3% in the control group (Fig. 1B,C). To determine the cell mortalities at the different time points of gp120 treatment, we treated neurons using 30 ng/mL gp120 for a series of time periods (0.5 h, 2 h, 4 h, 8 h, 16 h, 20 h, 36 h, 60 h and 76 h). The results showed that minor cell mortality (less than 15%) occurred when treated with gp120 (30 ng/mL) for shorter than 16 h, and a robust cell mortality was observed between 20 h and 36 h. Approximately 40% cell mortality occurred when treated for 24 h using 30 ng/mL gp120 for 24 h (Fig. 1D).

### p53 overexpression and translocation to the nucleus at a low dose of gp120 stress

**Figure 2:**
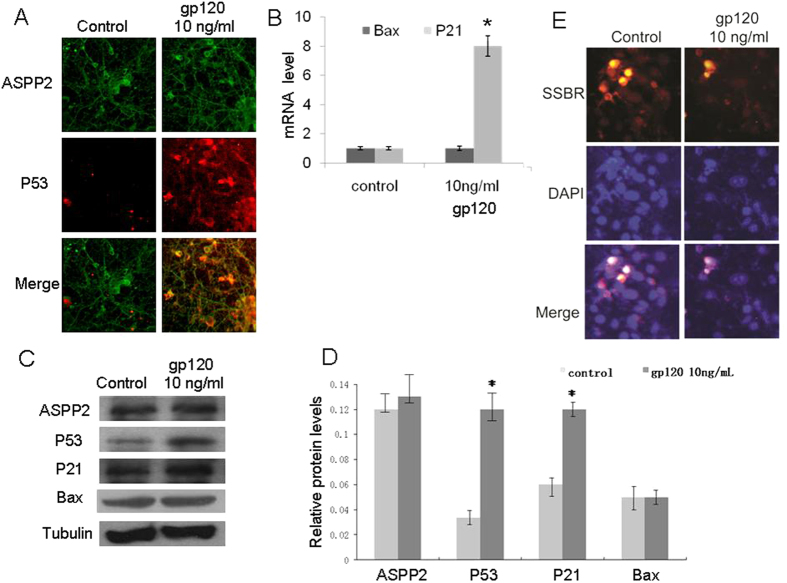
10 ng/mL gp120 for 6 hours induced p53 overexpression and translocation. (**A**) Immunofluorescence staining showed that p53, but not ASPP2, induced overexpression and translocation from the cytoplasm to the nuclei at 10 ng/mL gp120 for 6 hours. (**B)** qRT-PCR showed that only p21 overexpression was induced at 10 ng/mL gp120. (**C**) WB showed that only p21 overexpression was induced at 10 ng/mL gp120. (**D**) Relative levels of ASPP2, p53, p21 and Bax normalized with Tubulin. (**E**) Immunofluorescence staining showed that 10 ng/mL gp120 induced single-strand break repair. All data are shown as the mean ± SD of three independent experiments. *p < 0.05.

It has been reported that p21 can be upregulated by p53 and mediates the p53-dependent cell cycle G_1_ and S phase arrest in neurons when it was stressed by stroke or ischemia^22^,^23^. Here, we wanted to know the relationship of p53-associated gene expression and neuronal survival at a low dose of gp120 stress for 6 hours. First, we detected p53 and ASPP2 expression and the sublocation of these two proteins at a low dose of gp120 stress (Fig. 2A). The results showed that p53, but not ASPP2, induced overexpression and translocation from the cytoplasm to the nuclei at 10 ng/mL of gp120 for 6 hours. Then, p53 downstream genes Bax and p21 were detected. The results showed that only p21 induced overexpression at 10 ng/mL gp120 by qRT-PCR and WB, and Bax expression remained unchanged (Fig. 2B,C). These results suggested that p53 alone can stimulate p21 transcription. Finally, we detected single-strand break repair (SSBR) capability at 10 ng/mL gp120 stress. This result showed that neuronal death was not significantly different between the control and low dose gp120 stress groups. However, neuronal SSBR capability was significantly induced at a low dose of gp120 stress (Fig. 2D).

### Both p53 and ASPP2 translocate to the nucleus, but only p53 is overexpressed at a high dose of gp120 stress

**Figure 3:**
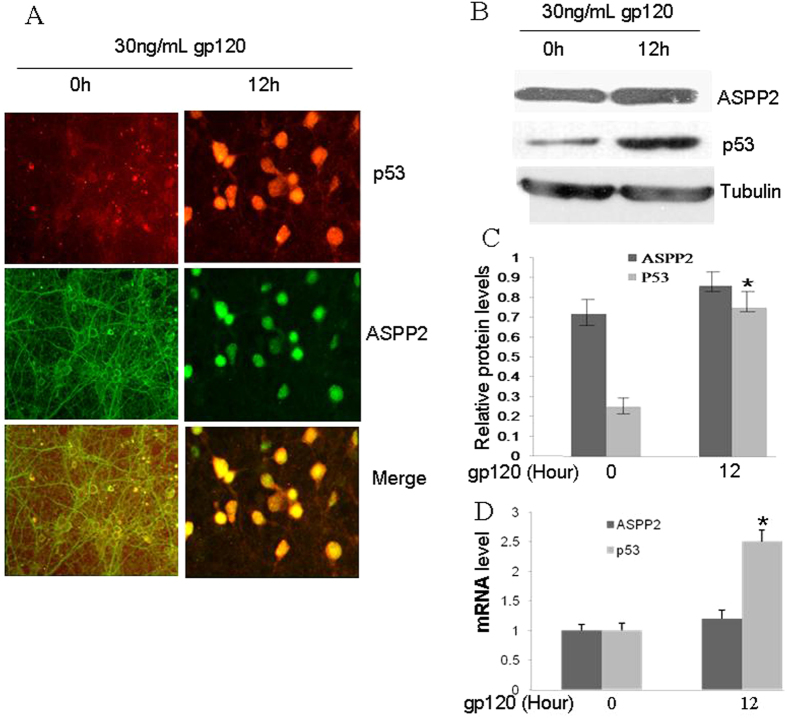
30 ng/mL gp120 for 12 hours induced both p53 and ASPP2 translocation to the nucleus and p53 overexpression. (**A**) Immunofluorescence staining showed that 30 ng/mL gp120 for 12 hours could stimulate ASPP2 to translocate from the cytoplasm to the nucleus as well as p53 overexpression and accumulation in nuclei. (**B**) WB revealed that 30 ng/mL gp120 for 12 hours only stimulated p53 protein overexpression. (**C**) Relative protein levels of ASPP2 and P53 normalized with Tubulin. (**D**) qRT-PCR showed that 30 ng/mL gp120 for 12 hours only stimulated p53 mRNA overexpression. All data are shown as the mean ± S.E.M. of three independent experiments. *p < 0.05.

It has been indicated that p53 could be overexpressed by gp120 and mediate the neuronal toxicity of gp120^24^. Here, we wanted to identify whether a high dose of gp120 can induce ASPP2 expression and subcellular location. First, we detected ASPP2 and p53 expression and subcellular location in cultured neurons at 30 ng/mL gp120 stress for 12 hours by immunofluorescence (Fig. 3A). From the results, we found that ASPP2 normally located in the cytoplasm and neurites of neurons, and 30 ng/mL gp120 for 12 hours could stimulate ASPP2 to translocate to the nucleus. Without stress, p53 was weakly expressed and its location could not be confirmed. Once treated with 30 ng/mL gp120 for 12 hours, p53 had significantly stimulated overexpression and accumulated in the nuclei. WB and qRT-PCR technology revealed that 30 ng/mL gp120 for 12 hours only stimulated p53 overexpression at both the mRNA and protein levels, and ASPP2 expression remained unchanged (Fig. 3B–D). These results suggest that high levels of gp120 can stimulate ASPP2 to translocate from the cytoplasm and neurites of neurons to the nuclei, but the expression levels of ASPP2 do not change.

### ASPP2-siRNA attenuated gp120-induced neuron death through inhibition of Bax expression

**Figure 4:**
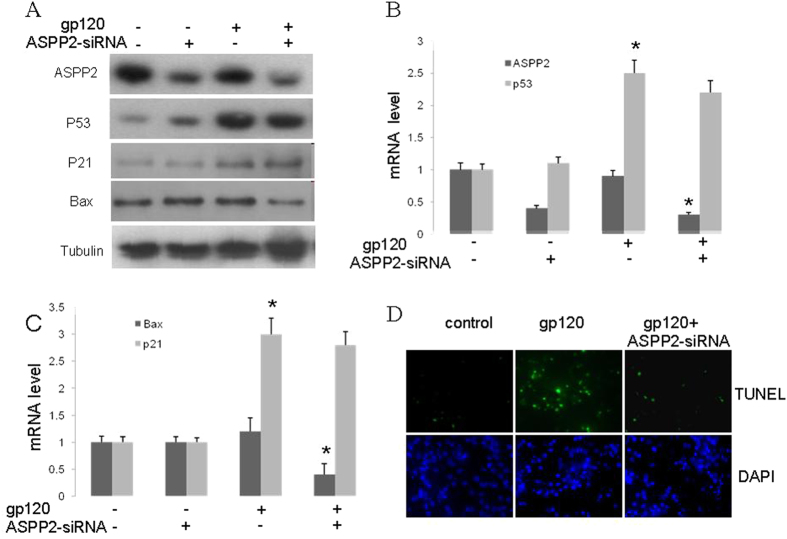
ASPP2-siRNA attenuated gp120 neurotoxicity by inhibition of Bax expression. (**A**) Bands of ASPP2, p53, p21 and Bax from neurons transfected by ASPP2 siRNA and then treated with gp120 for 12 h. WB showed that 30 ng/mL gp120 for 12 hours stimulated p53 but not ASPP2 overexpression, and ASPP2 and Bax expression could be attenuated by ASPP2-siRNA. (**B**) qRT-PCR revealed that 30 ng/mL gp120 for 12 hours only induced p53 mRNA overexpression, and ASPP2-siRNA could attenuate ASPP2 mRNA expression. (**C**) qRT-PCR showed that gp120 induced p21 mRNA overexpression, but ASPP2-siRNA decreased Bax mRNA levels. (**D**) The neuron apoptosis with ASPP2-siRNA treatment following gp120 stress. Data are shown as the mean ± SD of three independent experiments. *p < 0.05.

To confirm ASPP2’s involvement in p53-mediated neuron death, ASPP2 expression was shut down by ASPP2-siRNA. The ASPP2-siRNA sequence was designed as previous described^25^. The ASPP2-siRNA was delivered into cultured neurons through the Plentin virus vector (Invitrogen). After neurons were infected by ASPP2-siRNA-plentin for 48 hours, the ASPP2 expression could be detected with WB and qRT-PCR technology. The protein expression of p53 and ASPP2 were detected with WB in neurons at 30 ng/mL gp120 stress for 12 hours (Fig. 4A). The results showed that gp120 stimulated p53, but not ASPP2, overexpression, and ASPP2 expression could be attenuated by ASPP2-siRNA. Similarly, the qRT-PCR results revealed that gp120 only induced p53 mRNA overexpression and that ASPP2-siRNA could attenuate ASPP2 mRNA expression in neurons (Fig. 4B). Further, p21 and Bax transcription levels were detected with qRT-PCR and WB (Fig. 4C). It was revealed that ASPP2-siRNA attenuated gp120 associated Bax expression both in protein and mRNA levels, but p21 transcription was not affected. These results suggest that ASPP2 stimulates p53 transcription through the pro-apoptosis gene Bax but not cell cycle arrest gene p21. Neuron apoptosis was also investigated with ASPP2-siRNA treatment following gp120 stress (Fig. 4D). The results showed that ASPP2-siRNA significantly attenuated gp120 induced neuronal apoptosis.

### The higher survival rate of primary cultured p53^−/−^ASPP2^+/−^ neurons than p53^−/−^ neurons in gp120 exposure

**Figure 5:**
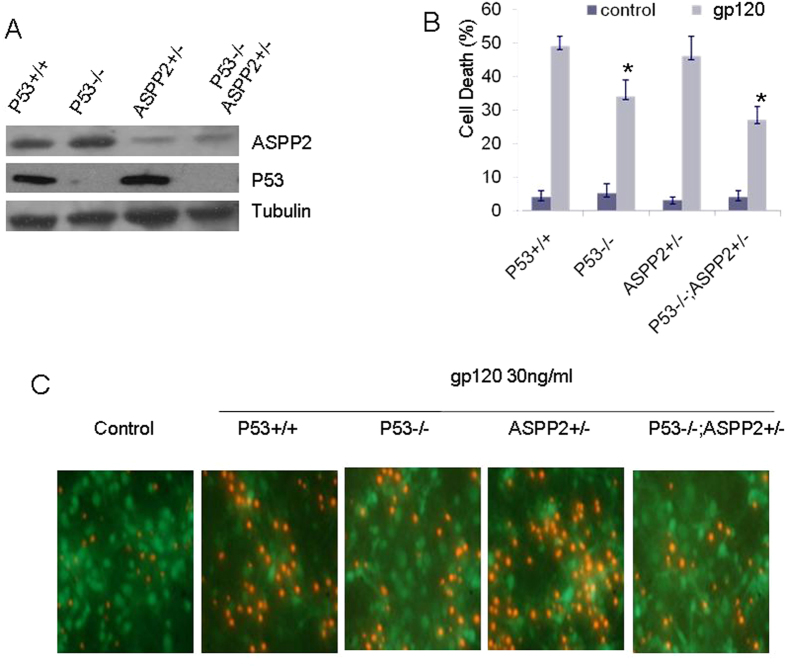
gp120 induced neuronal apoptosis in different p53 and ASPP2 genotype mice. (**A**) Mouse genotypes were identified by WB. (**B**) gp120-induced neuronal apoptosis in different mouse genotypes. (**C**) PI and Calcium double staining revealed gp120 induced neuronal apoptosis. Data are shown as the mean ± SD of three independent experiments. *p < 0.05.

ASPP2 can interact with p53 and induce cell apoptosis^25^, and p53 deletion will reduce the gp120-induced neurotoxicity and neuron apoptosis^24^. However, the roles of p53 and ASPP2 in gp120-induced neurotoxicity are still not clear. We first treated the primary cultured neurons of p53 and ASPP2 wild-type mice, p53^−/−^ mice and p53^−/−^ASPP2^+/−^ heterozygous mice with different concentrations of gp120 proteins, and neuron survival was analyzed with an MTT test. Mouse genotypes were identified by WB assay. Our results showed that the expression of ASPP2 in mice with ASPP2^+/−^ or P53^−/−^ASPP2^+/−^ genotype decreased significantly compared with the ASPP2 wild-type mice and p53^−/−^ mice (Fig. 5A). The results showed that the survival rate of gp120-treated p53^−/−^ASPP2^+/−^ neurons were significantly higher than that of p53^−/−^ neurons, and the survival rate of p53^−/−^ neurons was higher than that of wild-type p53/ASPP2 neurons (Fig. 5B). The PI and Calcium uptake test further confirmed that ASPP2 and p53 significantly induced gp120-induced neuronal death (Fig. 5C). The results suggest that both p53 and ASPP2 can mediate gp120-induced neuronal apoptosis.

### p53 interaction with ASPP2 induced by high dose of gp120 stimulated Bax transcription and contributed to caspase-3 cleavage

**Figure 6:**
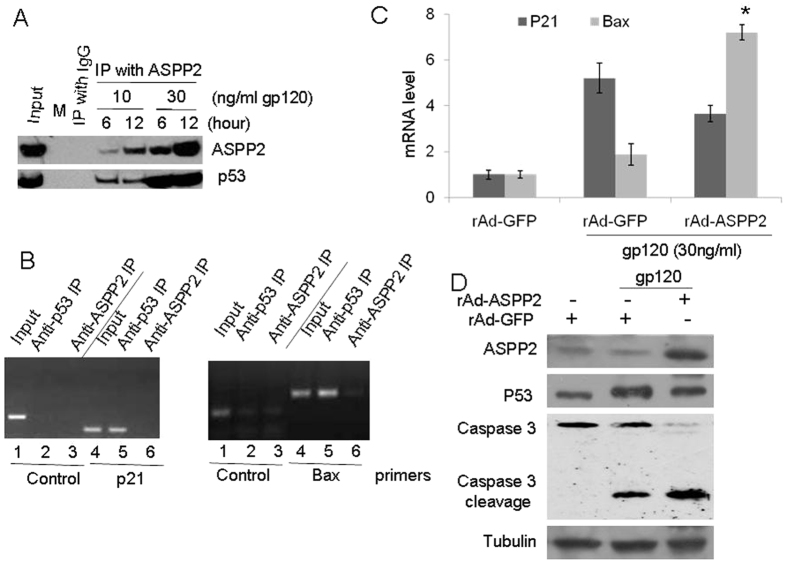
gp120 stress produced the interaction of p53 and ASPP2 and its downstream genes expression. (**A**) Immunoprecipitation showed that ASPP2 had a weak interaction with p53 at 10 ng/mL gp120 stress for 6 or 12 hours, and 30 ng/mL gp120 stress strongly induced the binding of ASPP2 and p53. (**B**) The results of ChiP-PCR with ASPP2 or p53 for immunoprecipitation and PCR targeted to the p53 binding motif in Bax and p21 promoter after treatment with 30 ng/mL gp120 for 6 hours. (**C**) rAD-ASPP2 technology showed that overexpressed ASPP2 significantly increased the transcription level of Bax induced by 30 ng/mL gp 120. (**D**) WB showed that more caspase 3 was cleaved into fragments in ASPP2- overexpressed neurons following by gp120 stress. Data are shown as the mean ± SD of three independent experiments. *p < 0.05.

ASPP2 interaction with p53 in DNA damage stress contributed to cell death^26^. We have previously confirmed that ASPP2 and p53 translocated to the nucleus following gp120 treatment. Now we could identify whether gp120 affected the interaction of ASPP2 and p53 and the regulatory function of p53. We first ran immunoprecipitation with anti-ASPP2 antibody at low and high doses of gp120 stress for 6 and 12 hours, and then ran WB with anti-p53 antibody. The results showed that ASPP2 had a weak interaction with p53 at low doses of gp120 stress for 6 and 12 hours, but high dose gp120 stress strongly induced the binding of ASPP2 and p53 (Fig. 6A). After cells were treated with a high dose of gp120 for 6 hours, ChiP-PCR with ASPP2 or p53 for immunoprecipitation and PCR targeted to the p53 binding motif in the Bax and p21 promoter showed that the weak PCR bands were detected on both p21 and Bax promoter targets when cell lysis was immunoprecipitated with anti-ASPP2 antibody (Fig. 6B). However, the strong PCR bands were identified at both p21 and Bax promoters following gp120 stress when cellular immunoprecipitation was conducted with anti-p53 antibody (Fig. 6B). Then, we treated the neuron with both rAD-ASPP2 and a high dose of gp120 and identified the role of overexpressed ASPP2 in neuronal death induced by gp120. The results showed that overexpressed ASPP2 did not affect the transcription level of p21 induced by a high dose of gp120 protein, but significantly increased the transcription level of Bax induced by high dose of gp120 protein (Fig. 6C). Bax is targeted to mitochondria and can induce cellular apoptosis via promoting cytocrome C release and sequenced activating caspase 3^27^. We identified the caspase 3 activation with WB, and the results showed that more caspase 3 was cleaved into fragments in ASPP2-overexpressed neurons following gp120 stress (Fig. 6D). These results suggest that ASPP2 plays an important role in p53-mediated cell death with gp120 stress.

## Discussion

Previous studies confirmed that HAND was associated with synaptic damage, disappearance of synaptic structure and neuronal apoptosis^28^. A large number of studies *in vivo* and *in vitro* suggest that gp120, the important functional protein in mediation of HIV fusion with target cells, has strong neurotoxicity and is an important pathogenic factor for HAND processes^29^,^30^. In the central nervous system (CNS), neuroinflammation mediated gp120 release from HIV-1 infected cells (macrophage or microglial) and activation of chemokine receptors can cause caspase-3, caspase-8 and caspase-9 dependent apoptosis, which is associated with the development of HAND^31^,^32^. Studies with cultured neurons have shown that the gp120-induced neuronal apoptosis only appeared in p53 wild-type neurons, but not in p53 homozygous deficient mouse neurons^33^, which was consistent with our present results. These results suggest that p53 may play an important role in neuronal apoptosis induced by gp120. Interestingly, research has indicated that gp120 may increase intracellular reactive oxygen species (ROS), but it also attenuated ROS-induced neuronal apoptosis via p53-mediated activation of neuronal autophagy^15^.

p53 is an important apoptosis-stimulating protein. In oxidative stress, hypoxia and other cellular stress situations, the p53 protein quickly upregulated expression and induced biological responses via transcriptional activation of specific target genes, such as Bax, p53-upregulated-modulator-of-apoptosis (PUMA) and others^34^. In addition, p53 also exerts its DNA repair and cell cycle arrest functions once activated by upstream signals, such as DNA damage. ASPP2 is a p53-binding protein that specifically stimulates the pro-apoptosis functions of p53. However, the role of ASPP2 on neuronal damage induced by gp120 has not been previously reported. In this study, we found that ASPP2 was located in the cytoplasm and did not confer apoptosis of neurons when treated with low doses of gp120, and cell cycle arrest may have occurred according to the increased expression of p53 and p21, and the increase in SSBR. The latter results coincided with the previous reports that the gp120 protein could regulate p53 phosphorylation and expression of MDM 2 and p21 in cultured neurons^24^,^35^.

Many apoptotic genes are sensitive to the regulation of the p53 gene including Fas, Fas ligand, Apaf1 (apoptosis protease-activating factor 1), MDM2, p21 and bcl-2 family members^24^,^36^. Recent studies showed that the function of p53 not only depends on p53 overexpression but also depends on post-translational modification including methylation, acylation, phosphorylation and ubiquitination^37^. The interaction of gp120 and CXCR4 could result in cellular oxidative damage, which consequently activated p53 and increased Apaf-1 phosphorylation that mediated neuronal apoptosis^38^,^39^. In this study, Bax, the bcl-2 family member, has been confirmed to be involved in p53-mediated neurotoxicity induced by gp120 in primary cultured mouse cortical neurons, and our study also showed that the neurotoxicity induced by high doses of gp120 is associated with the translocation of ASPP2 from the cytoplasm to the nucleus.

Although few opinions have been posed about whether or how ASPP2 was involved in the development of HAND, some research has been performed to confirm its function in neuropathy. ASPP2 plays a key role in controlling cell proliferation, polarity and tissue organization in the CNS^40^. The important role of ASPP2 in the development of Alzheimer’s disease through specific interaction with amyloid precursor protein (APP-BP1) has been reported^41^. Wilson confirmed that ASPP family members (ASPP1, ASPP2 and iASPP) have a specific regulatory function in the death of retinal ganglion cells after axonal injury and blockade of the ASPP-p53 pathway that is beneficial for central neuron survival^42^,^43^.

Our previous report with mouse anti-microtubule association protein-2 (MAP-2) antibody, which recognizes the neural cytoskeleton, indicated that gp120-treated cells appeared with a small cell body and thin, short, or even broken neuronal axons^10^. This study showed that the cultured neurons from different genotypes (wtp53, p53^−/−^ and p53^−/−^ASPP2^+/−^) had a different neuronal toxicity induced by gp120. More wild-type neurons died, and fewer of them had axon injures. Further, neuronal impairment was relatively milder in p53^−/−^/ASPP2^+/−^ double knockout mice. These results suggest that both ASPP2 and p53 are required for gp120-induced neuronal apoptosis.

In summary, our study showed that ASPP2 promoted neuronal apoptosis by inducing the expression of Bax and p53 in response to high doses of gp120; the underlying mechanism is promotion of the translocation of both ASPP2 and p53 to the nucleus and binding of the apoptosis-associated gene Bax. Further research is necessary to investigate the exact regulatory pathway of this process.

## Additional Information

**How to cite this article**: Liu, Z. *et al*. ASPP2 involvement in p53-mediated HIV-1 envelope glycoprotein gp120 neurotoxicity in mice cerebrocortical neurons. *Sci. Rep.* **6**, 33378; doi: 10.1038/srep33378 (2016).
